# Editorial: Adult functional (il)literacy: a psychological perspective

**DOI:** 10.3389/fpsyg.2024.1483889

**Published:** 2024-09-23

**Authors:** Katarzyna Chyl

**Affiliations:** International Projects Unit, Educational Research Institute, Warsaw, Poland

**Keywords:** low reading skills, adult literacy, adults with low literacy skills, adult readers, editorial

**Figure 1:**
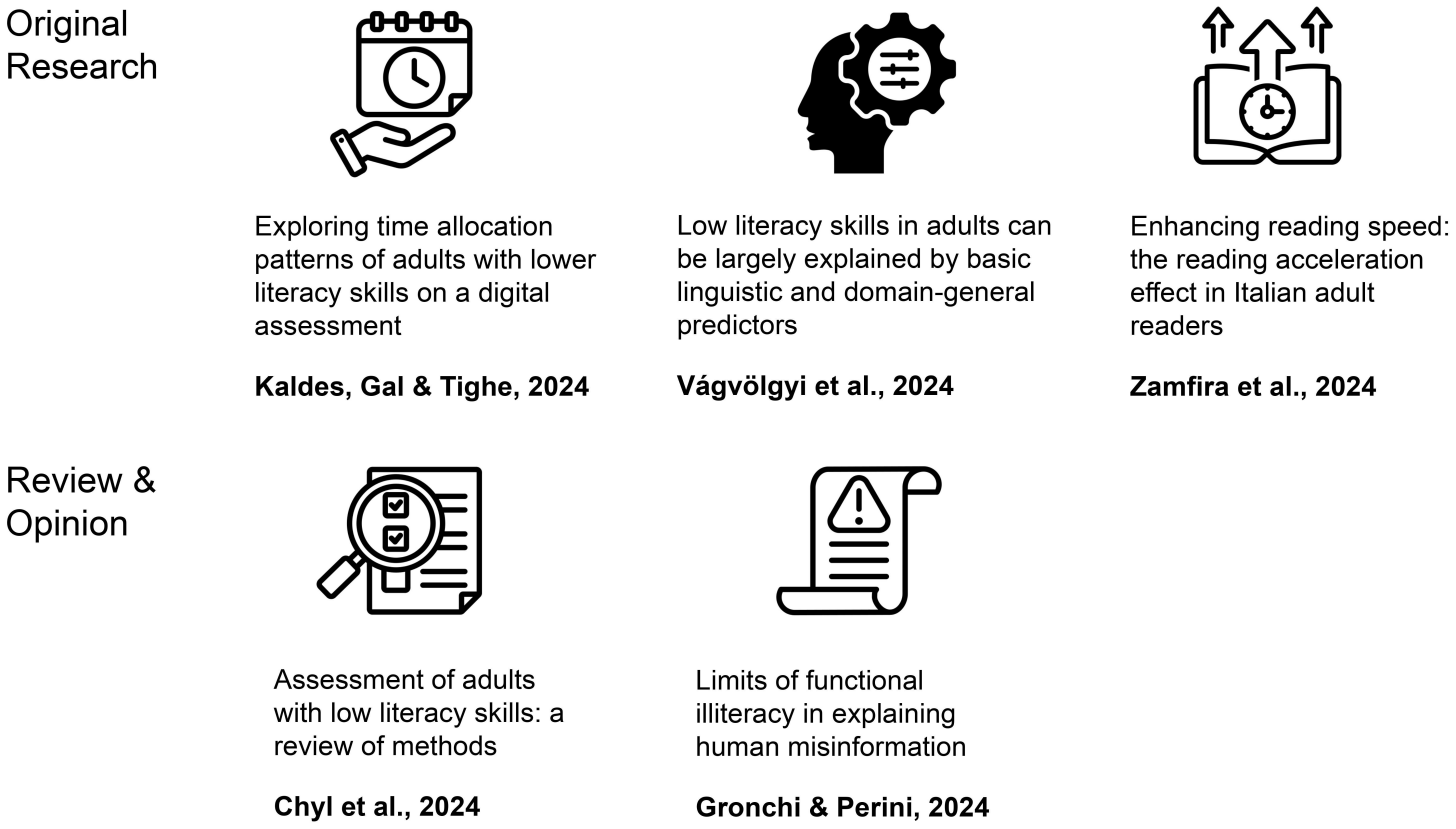
Articles collected in the Research Topic “*Adult functional (il)literacy: a psychological perspective*”. Designed by Freepik https://www.freepik.com/.

Learning to read is a crucial part of the primary education curriculum. However, reading is also indispensable outside the classroom and throughout adulthood. The inability to apply reading skills in everyday contexts is a severe obstacle to full participation in modern life. The purpose of this Research Topic was to collect the most recent articles focusing on adults with lower literacy skills. Calling for papers, we were interested in the cognitive underpinnings of reading skills, assessment ideas, and possible interventions. In total, we included five articles to the Topic, ranging from original research (Vágvölgyi et al.; Kaldes et al.; Zamfira et al.) to a review (Chyl et al.) and opinion papers (Gronchi and Perini), that will interest psychologists, educators, and other specialists. Noteworthy, our collection is not limited to studies performed in English, the language dominating the science of reading (Share, 2021). We have insights from different alphabetic scripts, varied in orthographic transparency. For the table of contents of our Topic, see Figure 1.

The use of the term ‘functional illiteracy' in the title of this research topic (and subsequently this editorial) has prompted a discussion about the language employed in both research and public discourse. While “functional illiteracy” traditionally refers to the inability to read and write at a level necessary for everyday tasks, its use can be problematic. The term carries the risk of stigmatizing individuals who struggle with reading, potentially reinforcing harmful stereotypes and marginalizing those who are already vulnerable. This discussion is similar to the debate on terms “dyslexia” and “dyslexics”, but the term “illiterate” is even less neutral. Nobody wants to be considered “illiterate”, regardless of the adjective. From the practical perspective, recruitment for research using this term can be difficult or even unethical, and communicating research results can be prone to misinterpretations (e.g., confusing “functional illiteracy” with “illiteracy”). We agree that more descriptive terminology (“adults with lower literacy skills”, “low literacy skills”) should become a new standard in the discipline and replace the formerly used term “functional illiteracy”.

Another problem with the term “functional illiteracy” discussed by Vágvölgyi et al., Gronchi and Perini, and Chyl et al., potentially inherited by the better term “lower literacy skill” is its operationalization. The definitions can vary greatly (Perry et al., 2017) or are not included in the papers at all (Perry et al., 2018). In the literature, the concept of “functional literacy” has also been extended to include, e.g., professional literacy or information literacy, which can be practical for researchers but further obscures the terminological chaos (Gronchi and Perini). The term “functional illiteracy” can also be tempting for researchers studying misinformation, as it seems to offer “simple explanations”, but the actual picture is much more complex (Gronchi and Perini). Low literacy skills, on the other hand, can mean both “decoding” and “practical skill use” depending on the context and research tradition (Chyl et al.). Defining the chosen term and selecting the appropriate assessment tools for the operational definition is essential. We strongly advise always to do that.

Two of the original articles in our Topic directly explore the cognitive underpinnings of low literacy skills. Kaldes et al. used publicly available data from the U.S. PIAAC study. The researchers analyzed time allocation patterns during test-taking (PIAAC's Level 2 and below). They found differences between proficiency levels and, importantly, a lot of heterogeneity within the group of adults with lower literacy skills. Divergent demographic profiles accompanied the two clusters of the fastest and slowest responders. As often only accuracy is examined in the studies on reading comprehension, much additional information goes unnoticed. The second original research comes from Vágvölgyi et al., who examined adult German speakers from basic education courses. The study investigated which linguistic, domain-general, or numerical factors predict reading performance and found that 73% of the variance can be explained by the combination of linguistic variables (decoding, oral semantic, and grammatical comprehension), working memory, and age. These results can drive interventions planned for adults with low literacy skills. In a semi-transparent orthography such as German, it could be helpful to focus on decoding, and both written and oral comprehension (Kindl and Lenhard, 2023).

An interesting addition to our Topic is an Italian study reporting a reading acceleration effect (Zamfira et al.). The protocol was based on previous Breznitz studies (e.g., Breznitz and Share, 1992). It relied on the assumption that readers can enhance their reading speed while maintaining high comprehension levels if they are forced to read faster than their usual reading rate. Even though all participants were typically reading university students, there was some variability in their reading speed, and slower readers showed the highest gains in reading speed. In this study, the enhanced reading did not compromise accuracy; however, only simple, local comprehension was measured, reaching the ceiling effect. The authors claim that this protocol may improve reading proficiency in different populations. It is time to check that also in adults with low literacy skills. Improvement in decoding efficiency in this group could support functional reading of everyday life texts.

Despite the importance of reading skills in the everyday life of adults in the modern world, the reading research is dominated by the school context, reading acquisition in young children, and problems caused by developmental dyslexia, especially in English. We believe that the articles collected in our Topic are a valuable addition to the discipline and move our understanding of low literacy skills forward.
